# Obesity and cognitive decline: role of inflammation and vascular changes

**DOI:** 10.3389/fnins.2014.00375

**Published:** 2014-11-19

**Authors:** Jason C. D. Nguyen, A. Simon Killcross, Trisha A. Jenkins

**Affiliations:** ^1^Discipline of Pharmaceutical Sciences, School of Medical Sciences, Health Innovations Research Institute, RMIT UniversityBundoora, VIC, Australia; ^2^School of Psychology, UNSW AustraliaSydney, NSW, Australia

**Keywords:** memory, hippocampus, cytokines, dementia, Alzheimer's disease, neuroinflammation

## Abstract

The incidence of obesity in middle age is increasing markedly, and in parallel the prevalence of metabolic disorders including cardiovascular disease and type II diabetes is also rising. Numerous studies have demonstrated that both obesity and metabolic disorders are associated with poorer cognitive performance, cognitive decline, and dementia. In this review we discuss the effects of obesity on cognitive performance, including both clinical and preclinical observations, and discuss some of the potential mechanisms involved, namely inflammation and vascular and metabolic alterations.

The incidence of obesity, classified by a body mass index (BMI, body mass divided by the square of one's height) >30 kg/m^2^, is rising steadily throughout the world's population. Attributed to unhealthy diets (that is over-consumption of food and beverages with a high content of fats, sugars, and salt) and physical inactivity, figures from the Organization for Economic Co-operation and Development 2014 Obesity report (OECD, 2014) suggest that worldwide 18% of the adult population are obese, with more than one in three adults in Mexico, New Zealand and the United States, and more than one in four in Australia, Canada, Chile, and Hungary included in this category.

Obesity can have damaging effects on many organ systems. Many of the comorbid conditions are related to metabolic syndrome, characterized by a large waist measurement, high triglyceride levels, glucose intolerance, and hypertension and thus risk factors for the development of non-insulin-dependent (type II) diabetes mellitus, systemic hypertension, coronary artery diseases, and heart failure. Moreover, the incidence of respiratory diseases such as obstructive sleep apnoea, gastrointestinal, and musculoskeletal disorders, thromboembolism, stroke and cancer are increased with obesity (Grundy, 2004; Haslam and James, 2005).

In addition, associations between obesity and impaired cognitive function, as well as risk of dementias such as Alzheimer's disease, have more recently been recognized. When we consider the growing population of overweight and obese people worldwide, along with an increasingly aging population, understanding the pathophysiology of obesity on the central nervous system and in particular those subregions important in learning, memory and executive functioning is essential. In this review we will focus on clinical evidence that obesity is associated with cognitive dysfunction and an increased risk of dementia, and complement this with preclinical data from animal models of excess weight gain and cognitive impairment. We will then discuss brain pathological changes that have been observed in these populations, focusing largely on brain regions important in learning and cognition, namely the hippocampus and frontal cortex, before ending with an assessment of the current understanding of dietary-induced systemic and central inflammation within these regions.

## Obesity and cognitive dysfunction

### Mild cognitive impairment

A growing body of research indicates that obesity in mid-life is a predictor of mild cognitive impairment at old age. Cognitive aging is a normal process where in older adulthood there is a structural and functional change that results in a deterioration of cognitive ability (Glisky, 2007). However, even when controlling for cognitive aging, studies show a negative correlation between BMI and global cognitive performance (Elias et al., 2005; Jeong et al., 2005; Hassing et al., 2010). A cross-sectional longitudinal study of over 2000 middle aged workers supported the linear association between BMI and cognitive function determined by the word-list learning test, which evaluates verbal learning and memory, and Digit-symbol Substitution test (DSST), which assesses attention, response speed, and visuomotor coordination. Obese people recalled fewer words from the list in the word-list learning test and took longer to complete DSST relative to normal weight individuals (Cournot et al., 2006). In another study combining ages from 20 to 82, overweight and obese people exhibited poorer executive function test performance than normal weight adults with no evidence of a BMI x age interaction (Gunstad et al., 2007). Across studies, the different cognitive domains analyzed make it difficult to draw absolute comparisons, but impairment of specific cognitive domains such as executive function and short-term memory have been consistently identified in obese individuals when compared to normal weight counterparts (Cournot et al., 2006; Mond et al., 2007; Lokken et al., 2009; Sabia et al., 2009).

### Dementia and Alzheimer's disease

Obesity is associated with not only an increased risk of development of mild cognitive impairment, but additionally, late-life dementia and Alzheimer's disease (Solfrizzi et al., 2004; Whitmer et al., 2005; Gustafson et al., 2012; Besser et al., 2014). The relative risk of the development of dementia and Alzheimer's disease for obese (BMI ≥ 30 kg/m^2^) and overweight (BMI = 25–29.9 kg/m^2^) individuals in midlife compared to normal weight individuals was 2.04 and 1.64, respectively (Anstey et al., 2011). Epidemiological studies have shown that obesity in middle age increases the risk of developing dementia and Alzheimer's disease, irrespective of associated medical conditions such as diabetes or vascular disease (Solfrizzi et al., 2004; Whitmer et al., 2005; Panza et al., 2010; Gustafson et al., 2012; Besser et al., 2014). For example Whitmer and colleagues reported that being overweight at age 40–45 increased ones risk of developing dementia by 35%, while being obese increased this risk to 74% when compared to normal weight individuals (Whitmer et al., 2005). The link of elderly obesity with dementia and Alzheimer's disease is complicated. Several studies have found an age dependent relationship with Alzheimer's disease and late-life obesity (Elias et al., 2003; Gustafson et al., 2003, 2009), while others have shown no or even negative correlations (Buchman et al., 2005; Stewart et al., 2005; Luchsinger et al., 2007; Fitzpatrick et al., 2009). A possible explanation of the confounding results is that weight loss is strongly associated with Alzheimer's disease and occurs before any presentation of cognitive impairment (Buchman et al., 2005; Stewart et al., 2005).

### Animal models of weight gain

Animal models, which allow for more accurate control of diet and other confounding factors than studies in humans, have also found that there is a detrimental effect of diet-induced obesity on cognition. Indeed, in high fat feeding models of obesity, impairments of working memory (Jurdak et al., 2008), learning (Molteni et al., 2002; Murray et al., 2009), and memory performance (Granholm et al., 2008; Kanoski and Davidson, 2010; Kosari et al., 2012) have been observed. A rodent study showed that consumption of a high fat diet (45%) for 3 months caused obesity, insulin resistance, and poor performance in the operant based delayed matching to position task examining short-term information retention and executive function (McNeilly et al., 2011). Furthermore, acquisition rates in learning have been observed to be impaired in rats fed a high fat diet (25%) for 3 months, as evaluated by radial arm water maze, where fat-fed rats took longer and made more errors trying to locate a hidden platform compared to control (Alzoubi et al., 2013). Interestingly, a further study has shown that rats fed a high fat diet (60%) for 3 months have impaired spatial memory that is independent of weight gain and blood pressure change (Kosari et al., 2012).

### Associations with neuropsychiatric illness

There is widespread prevalence of psychiatric symptomatology in individuals diagnosed with mild cognitive impairment and Alzheimer's disease (Lyketsos et al., 2002; Enache et al., 2011), and extensive comorbidity of psychiatric illness with obesity (Luppino et al., 2010; Megna et al., 2011). Medical issues and mobility restrictions associated with being overweight or obese can negatively impact on an individual's psychological well-being, and can lead to depression (Wardle and Cooke, 2005). In turn, mental health issues can lead to unhealthy lifestyle choices, such as diminished physical activity, increased appetite and poor food choices, smoking, and excessive alcohol intake (De Wit et al., 2010; Hoare et al., 2014). The use of psychiatric medicines, such as antipsychotics and antidepressants, to manage mental health issues in obese individuals may be problematic as there is a clear association between psychiatric medicines and significant weight gain (Reynolds and Kirk, 2010; Serretti and Mandelli, 2010; Hasnain et al., 2012). While many patients with a psychiatric illness are highly susceptible to cardiovascular disease, diabetes, and metabolic syndrome (De Hert et al., 2009; Pan et al., 2012), there is growing understanding of a role for hypothalamic-pituitary-adrenal axis dysfunction and basal systemic low-grade inflammation in the relationship between psychiatry and obesity. While this is beyond the scope of this review, recent researchers discuss the complex relationship between obesity and psychiatric illness (Hryhorczuk et al., 2013; Jaremka et al., 2013; Castanon et al., 2014; Miller and Spencer, 2014).

## Obesity and brain pathophysiology

The negative systemic effects of obesity on cardiovascular and metabolic physiology are well-recognized, and it is now clear that the brain is also negatively affected by obesity. Alterations in brain pathology of overweight/obese individuals who are otherwise healthy are supported by preclinical studies, demonstrating the possible underlying mechanisms by which obesity impairs higher cerebral function and exacerbates aging-related dementia remain wide and varied.

### Brain atrophy

Increased adiposity has been correlated with reduced volume in a number of brain regions. In a longitudinal study in a group of female patients born between 1908 and 1922, women with atrophy of the temporal lobe were found to have a higher BMI, with risk of temporal atrophy increased 13–16% per 1 kg/m^2^ BMI rise (Gustafson et al., 2004). More recent brain scanning techniques demonstrated that a group of obese individuals (BMI average 39) had significantly lower gray matter density in the post-central gyrus, frontal lobe, putamen, and middle frontal gyrus compared to a group of controls with a BMI of 22 (Pannacciulli et al., 2006). A further analysis in over 1400 Japanese healthy individuals revealed a significant negative correlation in men, though not in women, between BMI and brain gray matter ratio with temporal, occipital, and frontal lobes and the anterior lobe of the cerebellum showing reduced volume with increased BMI (Taki et al., 2008).

The hippocampal formation, a structure essential for learning and memory, is particularly susceptible to aging (Jack et al., 2000; Raji et al., 2009). It is also well-recognized that reduced hippocampal volumes predict cognitive decline and dementia in the general population (Elias et al., 2000; Amieva et al., 2005; Den Heijer et al., 2010). As we described previously, a majority of studies have found that obesity in mid-life is associated with an increased risk of developing dementia in later life, and consistent with this there is evidence from the Framingham Offspring Cohort Study of increased rates of hippocampal brain atrophy and executive function decline with mid-life obesity (Debette et al., 2011). However, this effect of obesity on hippocampal functioning is also found earlier: Adolescents with metabolic syndrome showed significantly smaller hippocampal volumes along with impaired attention and mental flexibility compared to non-obese children of similar ages (Yau et al., 2012).

Pre-clinical experimental rodent studies have also provided insight into the potential mechanisms underpinning obesity-related cognitive impairment. The affected cognitive domains involved in learning, memory, and executive function are mainly subserved by the hippocampus and prefrontal cortex. Long-term potentiation (LTP) is considered to be the major cellular mechanism that contributes to learning and memory where there is a synaptic change that leads to the formation of a stronger synapse (Bliss and Collingridge, 1993). In rodent models, high fat levels impair hippocampal LTP in the dentate gyrus (Karimi et al., 2013) and CA1 regions (Stranahan et al., 2008). Moreover a diet high in triglycerides was shown to diminish hippocampal long-term synaptic potential maintenance (Farr et al., 2008) suggesting a possible mechanism by which triglycerides mediate cognitive dysfunction associated with obesity.

At a cellular level, hippocampal changes are observed when diet is manipulated. Consumption of a high fat diet produces a reduction in molecules involved with neurogenesis, synaptic function and neuronal growth. A decrease in hippocampal neurogenesis in the dentate gyrus was observed after 4 weeks of feeding of a 42% fat diet (Lindqvist et al., 2006), while reduced levels of hippocampal markers of cellular proliferation (Kim et al., 2009) and hippocampal brain-derived neurotrophic factor (Molteni et al., 2002; Wu et al., 2003) have also been reported. Additionally consumption of dietary fats induces hippocampal (Rivera et al., 2013) and hypothalamic (Moraes et al., 2009) neuronal apoptosis and a reduction in hippocampal weight (Calvo-Ochoa et al., 2014) showing that high fat consumption impairs both new neuronal production and cell survival. It should be noted that not all diet manipulations have a negative effect on hippocampal function: Mice fed a diet rich in polyphenols and polyunsaturated fatty acids were observed to have more newly generated cells in the dentate gyrus (Valente et al., 2009).

Meanwhile in the prefrontal cortex reduced levels of dopamine (Geiger et al., 2008; Hansen et al., 2013) and acetylcholine (Morganstern et al., 2012) and increased markers of oxidative stress (Souza et al., 2007) have been observed in both obese-prone rat models and after high fat feeding, suggesting a dysfunction in this region which may contribute to associated observed behavioral deficits.

### Cerebrovascular

Vascular dementia is caused by cerebrovascular disease. Increasing evidence suggests that the vascular effects of obesity have a key role in the development of vascular cognitive impairment in aged people (Gorelick et al., 2011) by promotion of atherosclerosis in large cerebral arteries and alterations at the level of the cerebral microcirculation (Zlokovic, 2011). Indeed in a recent rodent study, mice fed a high fat diet displayed disruptions in cerebral vascular function including neurovascular coupling and functioning of arteries (Li et al., 2013; Lynch et al., 2013). Moreover, aging exacerbated obesity-induced decline in microvascular density in the hippocampus and cerebral cortex which was positively correlated with hippocampal-related cognitive function. Aging also exacerbated the obesity-induced oxidative stress and impaired cerebral blood flow indicating the possible effects of both aging and obesity and brain vascular integrity (Tucsek et al., 2014b).

### Alzheimer's disease related pathology

Amyloid plaques and neurofibrillary tangles containing tau protein are the pathological markers of Alzheimer's disease (Serrano-Pozo et al., 2011), accompanied by microglia activation and astrogliosis (Beach et al., 1989; Itagaki et al., 1989). Pathological progression is somewhat consistent with plaques, tangles, neuronal, and synaptic loss observed in medial temporal cortical regions such as entorhinal and perirhinal cortex, followed by hippocampus and cerebral cortex (National Institute on Aging, 1997). The mechanisms by which obesity influences risk of Alzheimer's disease remain to be fully understood. Higher levels of Amyloid-beta (Aβ, the main component of amyloid plaques) precursor protein (APP) and tau expression have been reported in hippocampal sections from morbidly obese patients without cognitive impairment, compared to a cohort of non-obese controls (Mrak, 2009). Indeed increased levels of plasma amyloid proteins have been found in a number of studies of obese individuals (Lee et al., 2009; Jahangiri et al., 2013) suggesting a possible mechanism linking midlife obesity with the later development of Alzheimer's disease.

A number of experimental studies have examined markers of Alzheimer's disease-related pathology in rodents receiving diets high in fat. Mice receiving a high fat diet displayed increased expression of amyloid precursor protein and APP processing enzyme (Thirumangalakudi et al., 2008; Puig et al., 2012) along with tau phosphorylation (Koga et al., 2014). Moreover in rats fed a high fat diet followed by streptozotocin injection to induce a model of type 2 diabetes, hippocampal APP-cleaving enzyme and Aβ were found to be present, and raised compared to controls, in the hippocampus (Zhang et al., 2009).

Similarly, diet-induced obesity has been shown to increase amyloid and tau pathology in transgenic mouse models of Alzheimer's disease. In the double-mutant presenilin (PS)-APP model just 7 weeks of diet modification resulted in both hypercholesterolemia and significantly increased levels of Aβ peptides in the brain that were strongly correlated with the levels of both plasma and brain total cholesterol (Refolo et al., 2000). Meanwhile, much longer dietary interventions, for example 10 months of a high fat (35%) formula to the triple transgenic (3xTg-AD) mice increased Aβ 40 and 42 concentrations and tau, suggesting that high-fat consumption promotes Alzheimer's disease-like neuropathology (Julien et al., 2010).

### Blood brain barrier

A functioning blood-brain barrier (BBB) has an important role in maintaining a precisely regulated microenvironment for reliable neuronal signaling by allowing entry into the central nervous system of essential nutrients and protecting the brain from blood-borne toxins (Ballabh et al., 2004). The chemical consequences of a high fat diet (including elevated fatty acids and sugars) may also influence the brain by disrupting the integrity of the BBB.

BBB dysfunction is associated with both Alzheimer's disease and vascular dementia (Skoog et al., 1998), and can be related to clinical vascular factors (Blennow et al., 1990). In a longitudinal study being overweight or obese in mid-life was correlated with lower BBB integrity almost a quarter of a century later (Gustafson et al., 2007). Further evidence is available from animal studies: Rats fed a Western diet for 3 months had a decrease in expression of tight junction proteins in the choroid plexus and BBB (Kanoski et al., 2010). Moreover, Western diet consumption in rats produces, as a consequence of this BBB dysfunction, increased permeability to a peripheral fluorescent tracer in the hippocampus (Kanoski et al., 2010; Davidson et al., 2012). Reduced BBB integrity and increased microgliosis in the hippocampus was also observed in rats fed a high-saturated-fat and cholesterol diet for 6 months (Freeman and Granholm, 2012) demonstrating that the hippocampus appears to be particularly vulnerable to diet-induced BBB disruption.

Mechanisms linking obesity to BBB dysfunction, and subsequent neuronal impairment, memory loss and dementia are not yet fully established. As stated previously obesity has been associated with increased levels of circulating plasma amyloid proteins (Lee et al., 2009; Jahangiri et al., 2013) and there is some suggestion that peripheral Aβ can impair BBB integrity by pathologically affecting the cerebrovasculature (Su et al., 1999). Further support for the relationship between obesity and degeneration of the BBB suggests that high circulating levels of fat impair active transport of consummatory regulatory hormones such as leptin and ghrelin through the BBB (Banks et al., 2004, 2008), perhaps inhibiting their positive roles in synaptic plasticity via actions in the hippocampus (Shanley et al., 2001; Diano et al., 2006). It should also be considered that obesity leads to increased circulatory inflammatory markers which in turn gain access to the hypothalamus by increasing BBB permeability and/or via areas that lack an effective BBB.

### Systemic inflammation

In obesity there is an accumulation of white adipose tissue which is the key site facilitating systemic inflammation (Odegaard and Chawla, 2013). Particularly, both hypertrophied adipocytes and adipose tissue-resident immune cells (primarily lymphocytes and macrophages) contribute to increased circulating levels of proinflammatory cytokines where there is an increase of tumor necrosis factor (TNF)-α, important feeding-related peptides such as leptin and resistin, plasminogen activator inhibitor 1, C-reactive protein and interleukins (IL)-1β and IL-6 (Visser et al., 1999; Yudkin et al., 1999; Ouchi et al., 2011) in obese individuals. Those with a higher waist circumference and waist-hip ratio also showed higher C-reactive protein and IL-6 concentrations, with IL-6 positively associated with total body fat (Hermsdorff et al., 2011) suggesting that these measures may be more highly correlated to inflammatory markers than increases in BMI (Hermsdorff et al., 2011; Thewissen et al., 2011).

Another mechanism by which chronic low grade inflammation occurs is through T-cells. A cross-sectional study of obese women found that T-cell derived cytokines (IL-23 and IL-17) were increased independent of increased abdominal fat and insulin resistance (Sumarac-Dumanovic et al., 2009). This has also been corroborated in a diet-induced obese mouse study (Winer et al., 2009). Obesity has also been shown to induce the accumulation and activation of macrophages in adipose tissue in both mice and humans (Weisberg et al., 2003; Xu et al., 2003; Drake et al., 2011).

Systemic inflammation can contribute to cognitive decline and dementia. The first functional link between obesity and inflammation was found in obese mice where adipose tissue was observed to secrete TNF-α (Hotamisligil et al., 1993). Further preclinical data have demonstrated that after a lipopolysaccharide (LPS) challenge in diet-induced obese rats, an exacerbated and prolonged fever was observed as well as an increase in plasma TNF-α, IL-6, and IL-1ra levels compared to lean controls (Pohl et al., 2009). Cytokines, such as IL-1β and IL-6 have been shown to disrupt neural circuits involved in cognition and memory (Jankowsky and Patterson, 1999; Gemma and Bickford, 2007). A recent meta-analysis identified that increased plasma levels of C-reactive protein and IL-6 is associated with an increase of dementia (Koyama et al., 2013). Elevated plasma IL-6 and IL-12 levels were also associated with impaired processing speed and executive function assessed via Stroop interference and digit symbol testing in a group of elderly participants between the ages of 70 and 90 (Trollor et al., 2012). Furthermore, an imaging study conducted by Harrison and colleagues showed that after inducing systemic inflammation by injection of *Salmonella typhi* vaccine an acute decline in spatial memory (but not medial temporal lobe independent procedural memory) was observed in humans (Harrison et al., 2014), suggesting that the medial temporal lobe is acutely sensitive to systemic inflammation.

### Central inflammation

Peripheral cytokines can act on the brain to induce local production of cytokines (Dantzer et al., 2008). As such, central inflammation is observed after high fat feeding and in genetic models of obesity, particularly in the hypothalamus (for review see Miller and Spencer, 2014). When we consider areas important in cognition, in db/db mice, a model of metabolic syndrome where obesity arises as a result of leptin receptor insensitivity (Hummel et al., 1966), IL-1β, TNF-α, and IL-6 mRNA expression levels in the hippocampus are increased when compared to wild type controls (Dinel et al., 2011). Moreover, in mice fed a 60% high fat diet for 20 weeks, raised TNF-α expression has been observed in the hippocampus (Jeon et al., 2012). Juvenile high fat diet intake did not influence basal expression of pro-inflammatory cytokines in the brain, but potentiated the enhancement of TNF-α expression specifically in the hippocampus after a peripheral immune challenge with LPS (Boitard et al., 2014). Chronic high fat diet consumption has also been shown to exacerbate LPS-induced cytokine mRNA expression of TNF-α and interferon-γ in the hippocampus as well as IL-6 and suppressor of cytokine signaling-3 in the hypothalamus (Andre et al., 2014). At this stage the prefrontal cortex is yet to be investigated.

### Microglia and astrocytes

Microglia, the primary mediators of the central nervous system's immune defense system release pro-inflammatory cytokines, chemokines, nitric oxide, and superoxide species (Loane and Byrnes, 2010). While the relationship between obesity-induced microglia expression within hypothalamic regions in animal models is well-documented (Miller and Spencer, 2014), new data indicate that brain regions involved in cognition and memory also show exacerbated microglial expression. In the db/db mouse, increased levels of microglial activation markers are observed throughout the hippocampus (Erion et al., 2014). Moreover in aged (24 months) mice, hippocampal microglial activation was shown to be exacerbated by 5 months treatment with a high fat diet (Tucsek et al., 2014a). In addition, treatment of cultured primary microglia with sera derived from these aged obese mice resulted in significantly more pronounced microglia activation and oxidative stress (Tucsek et al., 2014a).

Astrocytes are the most abundant glial cell within the central nervous system and respond to all forms of insults through a process referred to as reactive astrogliosis (Sofroniew and Vinters, 2010). Within the hypothalamus, astrocytes produce cytokines that drive inflammatory responses (Garcia-Caceres et al., 2013) although new data suggest central inflammation can extend beyond the hypothalamus in obesity regimes to affect areas directly related to cognition. Astrocytes from the CA3 region of hippocampus showed longer and less abundant projections in high fat diet mice (Cano et al., 2014). In obese Zucker rats a similar pathology is observed with a reported significant increase in the number of glial fibrillary acidic protein (GFAP)-immunoreactive astrocytes throughout all subfields of the hippocampus as well as frontal and parietal cortices (Tomassoni et al., 2013).

## Conclusion

It is abundantly evident that there is a deleterious effect of obesity/high fat feeding on cognitive performance. In human clinical studies, obesity has been shown to increase the risk of the development of mild cognitive impairment, in the form of short-term memory and executive function deficits, as well as dementia and Alzheimer's disease. Genetic and diet-induced models of obesity further support this link with obese animals displaying deficits in working memory, learning, and memory performance. The exact mechanisms or mediators that underlie the connections between obesity and the risk of cognitive impairment are still unknown but potential avenues of further research include brain atrophy, disruption in cerebrovascular function, development of Alzheimer's disease related pathology, BBB breakdown, and systemic and central inflammation.

Only a limited number of therapeutic options are currently available to treat dementia. These pharmaceutical agents have shown some potential to improve cognition but are effective for only some of the population, may be useful for only a limited time, and do not change the underlying disease process (Craig and Birks, 2005; Birks, 2006). Moreover, it is evident that obesity not only negatively impacts brain function and structure in adulthood and dementia, but clearly causes changes in the developing brain during childhood and adolescence (Liang et al., 2014), with researchers still coming to understand the long-term negative consequences of a life-time of being overweight on the brain. As such interventions that focus on education and life-style related factors to improve cognitive health (Lucke and Partridge, 2013) appear to be the most promising option. Increasing physical activity is certainly beneficial in many instances of cognitive decline as well as other neurological disturbances (Loprinzi et al., 2013) and may be the best treatment preference until the development of therapeutic options to treat cognitive deficits and/or prevent cognitive decline in obesity are available.

### Conflict of interest statement

The Guest Associate Editor Luba Sominsky declares that, despite being affiliated to the same institution as authors Jason C. D. Nguyen and Trisha A. Jenkins, the review process was handled objectively and no conflict of interest exists. The authors declare that the research was conducted in the absence of any commercial or financial relationships that could be construed as a potential conflict of interest.
